# Implementation of Evidence-Based Practice in Relation to a Clinical Nursing Ladder System: A National Survey in Taiwan

**DOI:** 10.1111/wvn.12076

**Published:** 2015-01-14

**Authors:** Yi-Hao Weng, Chiehfeng Chen, Ken N Kuo, Chun-Yuh Yang, Heng-Lien Lo, Kee-Hsin Chen, Ya-Wen Chiu

**Affiliations:** Department of Pediatrics, Chang Gung Memorial Hospital, Chang Gung University College of MedicineTaipei, Taiwan; Division of Plastic Surgery, Department of Surgery, Evidence-Based Medicine Center, Wan Fang Hospital and Department of Public Health, School of Medicine, Center for Evidence-Based Medicine, College of Medicine, Taipei Medical UniversityTaipei, Taiwan; Center for Evidence-Based Medicine, College of Medicine, Taipei Medical University; and Visiting Professor, Division of Preventive Medicine and Health Services Research, Institute of Population Health Sciences, National Health Research InstitutesTaipei, Taiwan; Department of Public Health, Kaohsiung Medical UniversityKaohsiung, Taiwan; Center for Evidence-Based Medicine, Taipei Medical UniversityTaipei, Taiwan; Department of Nursing, Wan Fang Hospital, Taipei Medical UniversityTaiwan; Evidence-Based Knowledge Translation Center, Wan Fang Hospital, Taipei Medical UniversityTaiwan; School of Nursing, College of Nursing, Taipei Medical UniversityTaiwan; Center for Evidence-Based Medicine, Taipei Medical UniversityTaipei, Taiwan; Graduate Institute of Clinical Medical Sciences, College of Medicine, Chang Gung UniversityTaiwan; Master Program in Global Health and Development, Health Policy and Care Research Center, College of Public Health and Nutrition, Taipei Medical UniversityTaipei, Taiwan

**Keywords:** evidence-based practice, advanced nurse, beginning nurse, clinical ladder, online database

## Abstract

**Background:**

Although evidence-based practice (EBP) has been widely investigated, few studies have investigated its correlation with a clinical nursing ladder system. The current national study evaluates whether EBP implementation has been incorporated into the clinical ladder system.

**Methods:**

A cross-sectional questionnaire survey was conducted nationwide of registered nurses among regional hospitals of Taiwan in January to April 2011. Subjects were categorized into beginning nurses (N1 and N2) and advanced nurses (N3 and N4) by the clinical ladder system. Multivariate logistic regression model was used to adjust for possible confounding demographic factors.

**Results:**

Valid postal questionnaires were collected from 4,206 nurses, including 2,028 N1, 1,595 N2, 412 N3, and 171 N4 nurses. Advanced nurses were more aware of EBP than beginning nurses (*p* < 0.001; 90.7% vs. 78.0%). In addition, advanced nurses were more likely to hold positive beliefs about and attitudes toward EBP (*p* < 0.001) and possessed more sufficient knowledge of and skills in EBP (*p* < 0.001). Furthermore, they more often implemented EBP principles (*p* < 0.001) and accessed online evidence-based retrieval databases (*p* < 0.001). The most common motivation for using online databases was self-learning for advanced nurses and positional promotion for beginning nurses. Multivariate logistic regression analyses showed advanced nurses were more aware of EBP, had higher knowledge and skills of EBP, and more often implemented EBP than beginning nurses.

**Linking Evidence to Action:**

The awareness of, beliefs in, attitudes toward, knowledge of, skills in, and behaviors of EBP among advanced nurses were better than those among beginning nurses. The data indicate that a clinical ladder system can serve as a useful means to enhance EBP implementation.

## INTRODUCTION

Clinical nursing ladder systems, defined as grading structures to facilitate career progression by defining different levels of clinical practice in nursing, have been well established for several decades (Bjork, Hansen, Samdal, Torstad, & Hamilton, 2007; Buchan 1999; Hsu, Chen, Lee, Chen, & Lai, 2005; Krugman, Smith, & Goode, 2000). In the past, clinical ladder systems were primarily based on the length of tenure to improve job satisfaction and staff retention (Buchan, 1999; Drenkard, & Swartwout 2005; Froman, 2001; Zimmer, 1972). At that time, the majority of hospitals delineated clinical competence of staff nurses through the clinical ladder system (Burket et al., 2010; Froman 2001; Fusilero et al., 2008; Hsu et al., 2005; Snyder, 1997). Clinical ladders can verify nursing competence and also promote excellence in nursing practice (Riley, Rolband, James, & Norton, 2009; Snyder, 1997). Such ladder systems allow nurses to advance based on their clinical knowledge and skills (Pettno, 1998). There are four practice levels of clinical nurses in this system. Newly employed nurses begin as novices (level I, N1). On completing probation and orientation, entry-level nurses advance to intermediate nurses (level II, N2). Intermediate nurses are capable of decision-making and have mastered technical nursing skills. After passing the examination for advancement, nurses become proficient at nursing (level III, N3). Finally, level III nurses advance to expert nurses (level IV, N4). The levels are differentiated according to qualitative and quantitative indicators, including clinical competence, professional excellence, and educational attainment (Froman, 2001). Overall, clinical ladder systems provide a means of establishing a practice-based system that differentiates nursing levels to facilitate professional development, improve staff relationships, reward competency, and enhance working motivation.

Evidence-based practice (EBP) is clinical practice based on scientific evidence, a clinician's expertise and a patient's values and preferences (Melnyk, Gallagher-Ford, Long, & Fineout-Overholt, 2014; Weng et al., 2014). It integrates clinical epidemiology, biostatistics, research methods and informatics into health care. EBP implementation mainly involves four steps (Weng, Kuo, Yang, Liao, et al., 2013): first, framing a clear question based on a clinical problem; second, verifying relevant evidence from the literature; third, critically appraising the validity of contemporary research; and fourth, applying the findings to clinical decision-making. Melnyk and Fineout-Overholt (2014) use a 7-step EBP process, including: Step 0: Cultivate a spirit of inquiry along with an EBP culture; Step 2: Ask the PICO(T) question; Step 3: Critically appraise the evidence; Step 4: Integrate the evidence with clinical expertise and patient preferences to make the best clinical decision; Step 5: Evaluate the outcome(s) of the EBP practice change; Step 6: Disseminate the outcome(s). EBP has been an important indicator for evaluating nursing competence (Watson, Stimpson, Topping, & Porock, 2002; Weng, Kuo, Yang, Lo, et al., 2013), and there now exists research-based EBP competencies for practicing nurses and advanced practice nurses that can be used to drive quality and safety of care (Melnyk, Gallagher-Ford, Long, & Fineout-Overholt, 2014).

## BACKGROUND

A number of individual factors are associated with implementation of EBP. In addition, clinical abilities are associated with levels of clinical ladders. Nevertheless, it is not clear whether EBP implementation is incorporated into clinical ladder systems (Bostrom, Rudman, Ehrenberg, Gustavsson, & Wallin, 2013). Studies attempting to evaluate the correlation of clinical ladders with EBP implementation are limited (Bjork et al., 2007; Buchan, 1999). Since EBP is a critical skill to improve healthcare quality and safety, it is imperative to understand EBP levels of staff nurses positioned within these four discrete clinical ladder levels.

In regional hospitals of Taiwan, a four-level ladder system has been widely adopted for more than 20 years (Hsu et al., 2005; Leu, Liao, Chang, & Su, 2010; Lin & Chen, 2004). Nurses who are willing to advance to the next level have to finish certain requirements (Lin & Chen, 2004). All levels of nurses provide direct patient care. However, investigation to clarify whether EBP is incorporated into the ladder system in Taiwanese hospitals is still lacking.

In the current survey, we attempted to identify correlations of clinical ladders with EBP implementation within national regional hospital settings in Taiwan. The current study could serve as a reference for improving dissemination of EBP.

## METHODS

### Design

A structured questionnaire was developed using questions based on our previously reported questionnaires (Chiu et al. 2010a; Chiu et al. 2010b; see Appendix S1, available with online version of this article). This study was conducted during a 4-month period in January to April 2011.

### Subjects

Targets of this study were staff nurses working in Taiwanese regional hospitals. A regional hospital is defined as a secondary care hospital, as appraised by Taiwan's Joint Commission of Hospital Accreditation. Cluster sampling was used to conduct the present study. Briefly, regional hospitals were divided into four clusters by location (northern, western, eastern, and southern Taiwan) and a random sample of each cluster was selected. Since there are more hospitals in northern and western Taiwan, we selected more hospitals in those areas. For the present study, we randomly enrolled 11 of the 65 regional hospitals in Taiwan, including three located in northern Taiwan, four in western Taiwan, and two each in eastern and southern Taiwan. Postal questionnaires were distributed to all registered nurses at the enrolled hospitals.

### The Study Questionnaire

The survey included items for measuring the profile of EBP—including the awareness of, beliefs in, attitudes toward, knowledge of, skills in, and behaviors of EBP. Questions related to perceptions (beliefs, attitudes, knowledge, and skills) of EBP were rated using a 5-point Likert scale (*strongly agree, agree, neutral, disagree*, and *strongly disagree*). EBP behaviors consisted of EBP implementation and use of evidence-based retrieval databases, which included three databases in Chinese and eight databases in English. The Chinese databases were the Index to Chinese Periodical Literature (ICPL), the National Digital Library of Theses and Dissertations in Taiwan (NDLTDT), and the Chinese Electronic Periodical Service (CEPS). The English databases were the Cumulative Index to Nursing & Allied Health Literature (CINAHL), the Cochrane Library, MD Consult, MEDLINE, ProQuest, UpToDate, Micromedex, and DynaMed. These 11 databases were selected because of their popularity. The ICPL and MEDLINE databases are freely accessible, while the other databases require payment from individuals or their organizations. Utilization was defined as access at least once per month during the previous 6 months. In addition, motivations for accessing the database were determined.

Background characteristics, including gender, age, teaching appointment, administrative position (defined as health professionals who are in charge of administrative affairs), working experience, level on the clinical ladder, and academic degree, were also examined. The clinical ladder was classified into four levels: N1, N2, N3, and N4. In this study, nurses were categorized into beginning (N1 and N2) and advanced (N3 and N4) nurses. Academic level was divided into five categories: (a) technical school (less than college) degree, (b) junior college (2-year university) degree, (c) bachelor's (4-year university) degree, (d) master's degree, and (e) doctorate.

### Validity and Reliability

Content validity was examined by 10 experts with more than 15 years of clinical experience. The internal consistency of all indexes was estimated using Cronbach's coefficient alpha. In this survey, a content validity index of 0.96 and Cronbach's coefficient alpha of 0.88 indicated sufficient validity and reliability of parameters in the questionnaire.

### Ethical Considerations

The study protocol was approved by the Ethical Review Board of the National Health Research Institutes. The questionnaire was accompanied by an introductory letter stating the purpose of this study and promising confidentiality. Return of the completed questionnaire was considered consent to participate in the study. All questionnaires were anonymous.

### Statistical Analyses

A 5-point Likert scale for the use of informational resources was dichotomized for further analyses. A self-rating report of either *strongly agree* or *agree* was regarded as a favorable answer, while the other three (*neutral*, *disagree*, and *strongly disagree*) were viewed as unfavorable answers. The statistical analyses were conducted using a commercially available program (SPSS 12.0 for Windows, SPSS, Chicago, IL, USA). Categorical variables were analyzed using a Chi-square test or Fisher's exact test when appropriate. Multivariate logistic regression model using the various demographic factors as the covariates was conducted to adjust for possible confounders. Odds ratio (OR) and 95% confidence intervals (CI) were expressed after adjusting for the control variables. Significance was defined as *p* < 0.05.

## RESULTS

### Demographic Data of Participants and Their Awareness of EBP

In total 6,707 questionnaires were distributed to nurses of enrolled hospitals, with 4,206 valid returns (for a return rate of valid questionnaires of 62.7%), including 2,028 N1, 1,595 N2, 412 N3, and 171 N4 nurses. Demographic data are summarized in Table 1 according to the level of the clinical ladder system. Most participants were female (98.8%). The mean age of all nurses was 31.1 years. Only a small minority (1.9%) of nurses had an advanced academic degree (master's or doctorate). In addition, 12.1% of nurses had a teaching appointment, and 7.2% had an administrative position.

**Table 1:** Demographic Data and Awareness of Evidence-Based Practice (EBP) Among 4,206 Registered Nurses in Taiwan

*n* (%)	Clinical ladder				*p*
	N1	N2	N3	N4	
**Gender**					0.201

Female	1,996 (98.4)	1,581 (99.1)	408 (99.0)	170 (99.4)	

Male	32 (1.6)	14 (0.9)	4 (1.0)	1 (0.6)	

**Age (years)**					<0.001

20∼25	732 (36.1)	93 (5.8)	0 (0)	0 (0)	

26∼30	682 (33.6)	643 (40.3)	61 (14.8)	12 (7.0)	

31∼35	385 (19.0)	535 (33.5)	155 (37.6)	46 (26.9)	

36∼45	191 (9.4)	275 (17.2)	164 (39.8)	85 (49.7)	

46∼65	38 (1.9)	49 (3.1)	32 (7.8)	28 (16.4)	

**Working experience (years)**					<0.001

< 5	1,239 (61.1)	318 (19.9)	32 (7.7)	10 (5.8)	

5∼10	576 (28.4)	860 (53.9)	135 (32.8)	35 (20.5)	

> 10	213 (10.5)	417 (26.1)	245 (59.5)	126 (73.7)	

**Academic level**					<0.001

Technical school	854 (42.1)	709 (44.5)	142 (34.5)	38 (22.2)	

Junior college	749 (36.9)	522 (32.7)	136 (33.0)	55 (32.2)	

Bachelor's	415 (20.5)	349 (21.9)	113 (27.4)	44 (25.7)	

Master's	10 (0.5)	15 (0.9)	21 (5.1)	33 (19.3)	

Doctorate	0 (0)	0 (0)	0 (0)	1 (0.6)	

**Faculty position**					<0.001

No	1,979 (97.6)	1,342 (84.1)	263 (63.8)	113 (66.1)	

Yes	49 (2.4)	253 (15.9)	149 (36.2)	58 (33.9)	

**Director**					<0.001

No	2,002 (98.7)	1,497 (93.9)	317 (76.9)	86 (50.3)	

Yes	26 (1.3)	98 (6.1)	95 (23.1)	85 (49.7)	

**Awareness of EBP**					<0.001

Yes	1,497 (73.8)	1,328 (83.3)	374 (90.8)	155 (90.6)	

No	531 (26.2)	267 (16.7)	38 (9.2)	16 (9.4)	


There were significant differences in age, working experience, academic level, and position (faculty or director) between advanced and beginning nurses (*p* < 0.001). Advanced nurses tended to have the following characteristics: they were older, had more working experience, had a higher academic degree, and were a faculty member or director.

Overall, 3,354 nurses (79.7%) were aware of EBP or related terms such as evidence-based medicine, evidence-based nursing, or evidence-based health care (Table 1). Advanced nurses were more likely to have heard about EBP than were beginning nurses (*p* < 0.001).

### Beliefs, Attitudes, Knowledge, and Skills of Participants

**Figure 1:**
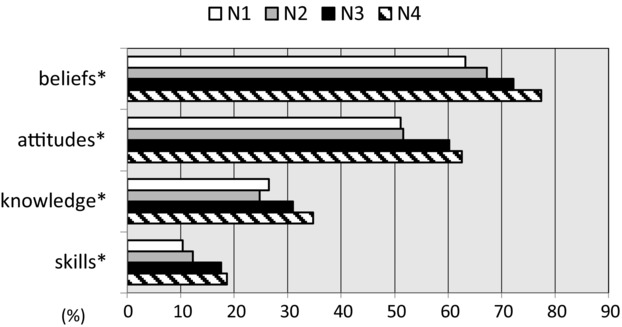
Beliefs in, attitudes toward, knowledge of, and skills in evidence-based practice (EBP) implementation. *Note*: *Beliefs – EBP is important for improving patient care quality. Attitudes – I am willing to support implementation of EBP. Knowledge – I have sufficient knowledge to implement EBP principles. Skills – I possess sufficient skills to implement EBP principles*.*p < 0.05 between beginning nurses (N1 + N2) and advanced nurses (N3 + N4).

Figure1 demonstrates the beliefs in, attitudes toward, knowledge of, and skills in EBP. Among 3,354 nurses who were aware of EBP, 2,229 nurses (66.5%) believed that EBP is important for improving patient care quality, and 1,772 respondents (52.8%) stated that they were willing to support EBP implementation. In addition, 855 (25.5%) nurses rated their knowledge sufficient to implement EBP, and 414 (12.3%) perceived that they had sufficient skills to implement EBP.

There were significant discrepancies in the EBP beliefs, attitudes toward EBP, knowledge of EBP, and skills in EBP between advanced and beginning nurses. Advanced nurses were more likely to have positive beliefs in EBP (*p* < 0.001), attitudes toward EBP (*p* = 0.001), knowledge of EBP (*p* = 0.008), and skills in EBP (*p* = 0.001) than were beginning nurses.

### Behaviors of EBP

#### Implementation of EBP

**Figure 2:**
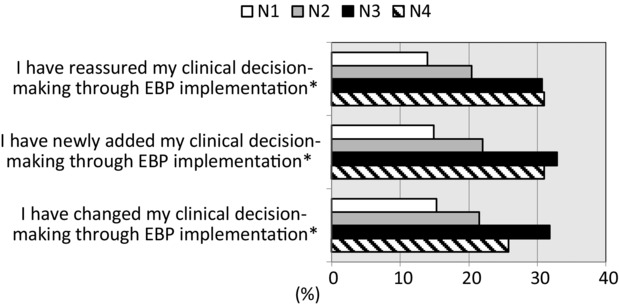
Implementation of evidence-based practice (EBP) by the clinical ladder nursing system. *Note*: *p < 0.05 between beginning nurses (N1 + N2) and advanced nurses (N3 + N4).

Among 3,354 nurses who were aware of EBP, 1,231 respondents (36.7%) had implemented EBP for clinical decision-making in the past year. There was a significant difference in EBP implementation among the four levels of the clinical ladder (Figure2). Advanced nurses more often changed, newly added, or reassured their clinical decision-making through EBP implementation than beginning nurses (*p* < 0.001).

#### Access to evidence-based retrieval online databases

**Figure 3:**
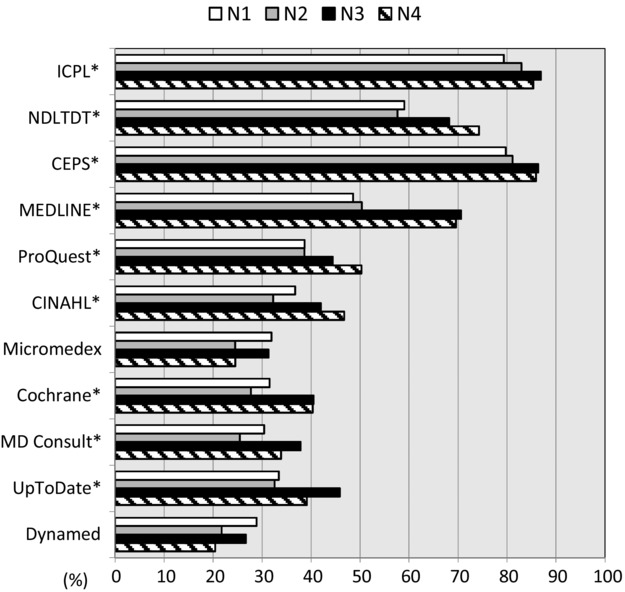
Access to 11 evidence-based retrieval online databases. *Note*: *p < 0.05 between beginning nurses (N1 + N2) and advanced nurses (N3 + N4).

The use of evidence-based retrieval online databases is illustrated in Figure3. In general, all levels of nurses preferred accessing databases in Chinese. There were significant differences in the use of databases between advanced and beginning nurses. Advanced nurses more often accessed nine databases (ICPL, NDLTDT, CEPS, MEDLINE, ProQuest, CINAHL, Cochrane Library, MD Consult, UpToDate) than did beginning nurses (*p* < 0.001).

**Figure 4:**
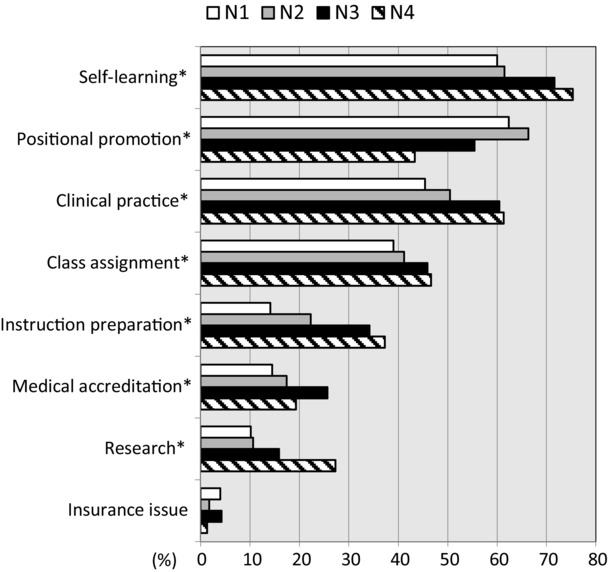
Motivation to access evidence-based retrieval databases. *Note*: *p < 0.05 between beginning nurses (N1 + N2) and advanced nurses (N3 + N4).

The motivations to access evidence-based retrieval online databases are demonstrated in Figure4. Overall, the most common motivation was self-learning (62.4%), followed by positional promotion (62.3%), clinical practice (49.5%), class assignment (40.9%), instruction preparation (20.3%), medical accreditation (17.0%), research (11.7%), and insurance issues (3.1%). For advanced nurses, the most common motive for using online databases was self-learning (72.7%). In contrast, the most common motive for beginning nurses was positional promotion (64.1%). There were significant differences in these motivations among the four groups of nurses. Compared to beginning nurses, advanced nurses more often rated self-learning (*p* < 0.001), clinical practice (*p* < 0.001), class assignment (*p* < 0.001), instruction preparation (*p* < 0.001), medical accreditation (*p* < 0.001), and research (*p* < 0.001) as motivations to access online databases. In contrast, beginning nurses more often reported positional promotion as the motivation compared to advanced nurses (*p* < 0.001).

### EBP According to Work Experience Among Beginning and Advanced Nurses

**Figure 5:**
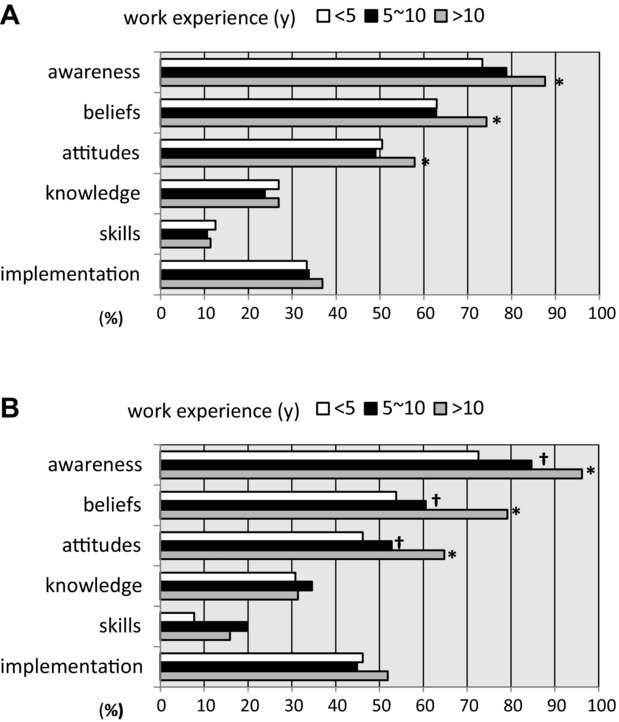
EBP of registered nurses according to work experience. (A) Beginning nurses (N1 + N2).(B) Advanced nurses (N3 + N4). *Note*: *p < 0.05 vs. work experience 5∼10 y and <5 y; ^†^p < 0.05 vs. work experience <5 y.

The association of EBP with work experience by the levels of ladder system is shown in Figure5. Among both beginning and advanced nurses, the awareness, beliefs, and attitudes of nurses with work experience of greater than 10 years were significantly more favorable than those with work experience of 5∼10 years and less than 5 years. In addition, the awareness, beliefs, and attitudes of advanced nurses with work experience of 5∼10 years were significantly more favorable than advanced nurses with work experience of less than 5 years.

### Analyses by Multivariate Logistic Regression Model

The demographic factors—including age, education, work experience, director, and faculty position—were incorporated into the multivariate logistic regression analyses (Table 2). In this multivariate model, advanced nurses were more aware of EBP (*OR* = 1.523, 95% *CI* = 1.086 ∼2.135), had more sufficient EBP knowledge (*OR* = 1.394, 95% *CI* = 1.101∼1.767) and skills (*OR* = 1.440, 95% *CI* = 1.060∼1.957) in EBP, and more often implemented EBP (*OR* = 1.473, 95% *CI* = 1.178∼1.841) than beginning nurses. Beliefs in and attitudes toward EBP were not significantly associated with the ladder system in the multivariate logistic regression model.

**Table 2:** Evidence-Based Practice (EBP) in Relation to Clinical Ladder System (Advanced Nurses vs. Beginning Nurses) Analyzed by Multivariate Logistic Regression Model

EBP	OR	95% CI	*p* value
Awareness	1.523	1.086–2.135	0.015

Beliefs	1.123	0.829–1.521	0.453

Attitudes	0.994	0.750–1.317	0.966

Knowledge	1.394	1.101–1.767	0.006

Skills	1.440	1.060– .957	0.020

Implementation	1.473	1.178–1.841	0.001


Covariates: age, education, work experience, director, and faculty position.

## DISCUSSION

In this study, we examined how EBP is perceived among nursing practitioners in regional hospitals nationwide in Taiwan. This national study allowed us the opportunity to compare and contrast various levels of EBP awareness, beliefs, attitudes, knowledge, skills, and behaviors between different levels of the clinical ladder. The findings indicate that levels of the clinical nursing ladder system reflect competence of EBP. To our knowledge, this is the first study to systematically investigate how nurses implement EBP according to the clinical ladder system.

Our study verified significant discrepancies in EBP between advanced and beginning nurses. First, advanced nurses were more aware of EBP than beginning nurses. This is probably because EBP has not yet been incorporated into nursing curricula (Chiu et al., 2010b). A majority of nurses learn about EBP when they practice in hospitals. Second, univariate analysis indicated that advanced nurses were more likely to have favorable beliefs about and attitudes toward EBP. Familiarity with EBP may be the possible reason that advanced nurses better recognize the value of EBP (Gerrish et al., 2012; Sandstrom, Borglin, Nilsson, & Willman, 2011). Nevertheless, our results by multivariate analysis did not show significant differences in beliefs and attitudes between the beginning and advanced nurses.

Third, advanced nurses had more sufficient knowledge and skills than beginning nurses. We speculated that the clinical ladder system can provide a way of encouraging continuing education for updating one's professional skills. Fourth, advanced nurses more often implemented EBP than beginning nurses. It is possible that clinical roles between advanced and beginning nurses are divergent. Advanced nurses need to find the best-quality evidence and make clinical decisions for the most effective health care. In contrast, beginning nurses may rather rely more on clear directions and may have less discretion in nursing decision-making. In addition, advanced nurses were more likely to serve as a leader in the workplace or as a faculty member. Some nursing characteristics were related to EBP implementation, such as having a leadership or faculty position (Gifford, Davies, Edwards, Griffin, & Lybanon, 2007; Sandstrom et al., 2011; Weng, Kuo, Yang, Lo, et al., 2013). Leadership was identified as an important individual factor for EBP implementation (Bostrom et al., 2013; Gifford et al., 2007; Sandstrom et al., 2011).

Fifth, registered nurses at advance levels tended to access evidence-based databases. This finding is in accordance with a survey in Norway showing that advanced nurses more often looked for information in databases (Bjork et al., 2007). Our study further extends their inquiry by illustrating an increase in use of a variety of databases among advanced nurses, including databases in Chinese and English. Online databases are increasingly being used as key resources to search for information with a summary of individual research evidence (Weng, Kuo, Yang, Lo, Shih, et al., 2013). It is imperative to disseminate EBP implementation to nurses by expediting their efforts to access evidence-based online databases. In this study, nurses were less apt to rely on online databases in English than those in Chinese. The data are consistent with our previous reports showing that language is an important barrier for nurses in Taiwan (Chiu et al., 2012; Weng, Kuo, Yang, Lo, Shih, et al., 2013). The main reason is that nurses customarily write nursing records in Chinese. In addition, the current study found that advanced nurses are more likely to access online databases than beginning nurses. Furthermore, the majority of information accessed from online databases was for professional purposes and positional promotion. The findings lead to the suggestion of using the clinical ladder system as a promotional means to enhance EBP implementation.

Overall, the majority of nurses recognize the value of EBP. However, most nurses lack methodological competence in EBP. The low levels of knowledge of and skills in EBP are consistent with previous reports showing that nurses are not ready for EBP (Adib-Hajbaghery, 2009; Chiu et al., 2010a; Melnyk et al., 2004; Thorsteinsson, 2013). A main contributing factor to this lack of knowledge and skills is that many academic programs continue to teach the rigorous process of conducting research instead of how to take an evidence-based approach to care (Melnyk et al., 2014). Integrating EBP throughout nursing curricula is necessary to prepare graduates with these skills who can accelerate its implementation (Chiu et al., 2010b).

Although the levels of the clinical ladder were significantly associated with work experience in this study, our analyses did not show any difference in the knowledge of, skills in, and implementation of EBP between longer and shorter work experience. Nevertheless, we found nurses with longer work experience hold more favorable awareness of, beliefs in, and attitudes toward EBP than those with shorter work experience. These data suggest that nursing environment in the hospital setting might provide certain support for EBP. Therefore, nurses will change their perceptions to EBP with time. Continuing education for the implementation of EBP may be required to increase their knowledge of and skills in EBP.

**LINKING EVIDENCE TO ACTION**

- EBP has been successfully incorporated into a clinical nursing ladder system. In an attempt to facilitate its spread, policy makers should use promotion as a motive to encourage nurses for advanced levels.
- Advanced nurses achieved higher competencies in EBP. They can serve as early adopters to speed up the dissemination of EBP.
- Integrating EBP into academic educational programs is necessary to accelerate EBP implementation.
- To enhance the knowledge of and skills in EBP, continuing education aiming to implement EBP is necessary.

## LIMITATIONS

There are several limitations to this study. First, this was a self-administered survey, not an audit of actual practice. Therefore, the results might not reflect the realities of practice under routine clinical care. Second, inaccuracies may have occurred in the questionnaire survey; however, there is no other reliable method for collecting such data on a nationwide basis. Third, the return rate of this questionnaire survey was 59.1%; however, we believe our respondents are a representative sample because their backgrounds were similar to those in our previous surveys.

## IMPLICATIONS

The clinical ladder system has been used in the nursing field for a very long time. However, the relationship between the ladder system and EBP has not been clearly surveyed. The current study indicates that the clinical ladder system has played an important role in accelerating EBP implementation. These data will provide critical evidence to guide strategies for finding relevant information to improve the effectiveness of EBP implementation.

## CONCLUSIONS

Our survey presents two potentially useful findings. First, this is the first survey to assess the benefits of the clinical ladder system in implementing EBP. Second, our study represents a nationwide sample and can be generalized to similar hospital settings. The clinical advancement program used in Taiwan's regional hospitals is associated with the EBP profile of staff nurses—including their awareness, beliefs, attitudes, knowledge, skills, and behaviors. The data demonstrate that the clinical ladder system reflects levels of EBP competence. In conclusion, EBP has been adopted as a core competence in the clinical ladder system for promotion. The data suggest that curricular programs of EBP are necessary for nursing schools in Taiwan to prepare graduates with excellent EBP knowledge and skills and facilitate its spread throughout clinical practice settings.

## SUPPORTING INFORMATION

Disclaimer: Supplementary materials have been peer-reviewed but not copyedited.

**Appendix S1**. Questionnaire Survey.
