# Supplementary material for: Implementation of Evidence-Based Practice in Relation to a Clinical Nursing Ladder System: A National Survey in Taiwan
Source: Worldviews Evid Based Nurs. 2015 Jan 14;12(1):22–30. doi: 10.1111/wvn.12076 (PMC4345401; doi:10.1111/wvn.12076)
Supplement: Supplementary file 1 — Appendix S1. Questionnaire Survey. [file wvn0012-0022-sd1.docx]

**Appendix**

**Questionnaire Survey**

1. **Main questions**
   1. Have you heard of EBP (evidence-based practice) or related terms, such as EBM (evidence-based medicine) and EBN (evidence-based nursing)?
      - yes (please continue)
      - no **(please skip to Section B)**
   2. Please rate your perception of the following questions.

|  | strongly agree | agree | neutral | disagree | strongly disagree |
| --- | --- | --- | --- | --- | --- |
| EBP is important for improving patient care quality | □ | □ | □ | □ | □ |
| I am willing to support implementation of EBP | □ | □ | □ | □ | □ |
| I have sufficient knowledge to implement EBP principles | □ | □ | □ | □ | □ |
| I possess sufficient skills to implement EBP principles | □ | □ | □ | □ | □ |

- 1. I have implemented EBP for clinical decision-making in the past year.
     - yes (please continue)
     - no **(please skip to Question 4)**

How do you implement EBP after literature searching and critical appraisal?

|  | Yes | No |
| --- | --- | --- |
| I have changed my clinical decision-making through EBP implementation | □ | □ |
| I have newly added my clinical decision-making through EBP implementation | □ | □ |
| I have reassured my clinical decision-making through EBP implementation | □ | □ |

- 1. How many times per month have you accessed the following evidence-based retrieval online databases to search medical information during the previous 6 months?

| average times | >12 | 9-12 | 5-8 | 1-4 | 0 |
| --- | --- | --- | --- | --- | --- |
| Index to Chinese Periodical Literature (ICPL) | □ | □ | □ | □ | □ |
| National Digital Library of Theses and Dissertations in Taiwan (NDLTDT) | □ | □ | □ | □ | □ |
| Chinese Electronic Periodical Service (CEPS) | □ | □ | □ | □ | □ |
| Cumulative Index to Nursing & Allied Health Literature (CINAHL) | □ | □ | □ | □ | □ |
| Cochrane Library | □ | □ | □ | □ | □ |
| MD Consult | □ | □ | □ | □ | □ |
| MEDLINE | □ | □ | □ | □ | □ |
| ProQuest | □ | □ | □ | □ | □ |
| UpToDate | □ | □ | □ | □ | □ |
| Micromedex | □ | □ | □ | □ | □ |
| DynaMed | □ | □ | □ | □ | □ |

- 1. What is (are) your motivation(s) to access evidence-based retrieval databases? (multiple choice)

|  | **Motivation** |
| --- | --- |
| □ | Class assignment |
| □ | Clinical practice |
| □ | Instruction preparation |
| □ | Self-learning |
| □ | Positional promotion |
| □ | Research |
| □ | Insurance issue |
| □ | Medical accreditation |

1. **Personal Information**
   1. Gender: □ male □ female
   2. Birth year: ________
   3. Academic degree
       □ technical school
       □ junior college
       □ bachelor’s
       □ master’s
       □ Ph.D.
   4. Your clinical ladder:
       □ N1
       □ N2
       □ N3
       □ N4
   5. How long have you worked since obtaining your license? ______ year(s)
   6. Are you currently a faculty member?
       □ yes □ no
   7. Are you currently a director in clinical service?
       □ yes □ no

**Thank you very much for your time and cooperation!!**
